# Population genetics of *Plasmodium falciparum* and *Plasmodium vivax* and asymptomatic malaria in Temotu Province, Solomon Islands

**DOI:** 10.1186/1475-2875-12-429

**Published:** 2013-11-22

**Authors:** Karen-Ann Gray, Simone Dowd, Lisa Bain, Albino Bobogare, Lyndes Wini, G Dennis Shanks, Qin Cheng

**Affiliations:** 1 Drug Resistance and Diagnostics, Australian Army Malaria Institute, Weary Dunlop Drive, Gallipoli Barracks, Enoggera, QLD, 4051, Australia; 2 Pacific Malaria Initiative Support Centre, School of Population Health, University of Queensland, Herston, QLD, Australia; 3 Malaria Drug Resistance and Chemotherapy, QIMR Berghofer Medical Research Institute, Herston, QLD, Australia; 4 Vector Borne Disease Control Programme, Ministry of Health, Honiara, Solomon Islands

## Abstract

**Background:**

Temotu Province, Solomon Islands is progressing toward malaria elimination. A baseline survey conducted in 2008 showed that most *Plasmodium* infections in the province were of low parasite density and asymptomatic infections. To better understand mechanisms underlying these malaria transmission characteristics genetic diversity and relationships among *Plasmodium falciparum* and *Plasmodium vivax* populations in the province were examined.

**Methods:**

Forty-five *P. falciparum* and 67 *P. vivax* samples collected in the 2008 baseline survey were successfully genotyped using eight *P. falciparum* and seven *P. vivax* microsatellite markers. Genetic diversity, relationships and distribution of both *P. falciparum* and *P. vivax* populations were analysed.

**Results:**

*Plasmodium falciparum* population exhibited low diversity with 19 haplotypes identified and had closely related clusters indicating clonal expansion. Interestingly, a dominant haplotype was significantly associated with fever and high parasite density. In contrast, the *P. vivax* population was highly diverse with 58 haplotypes identified that were not closely related. Parasite populations between different islands in the province showed low genetic differentiation.

**Conclusion:**

The low diversity and clonal population of *P. falciparum* population may partially account for clinical immunity developed against illness. However, it is possible that importation of a new *P. falciparum* strain was the major cause of illness. High diversity in *P. vivax* population and low relatedness between strains suggested clinical immunity to *P. vivax* may be maintained by different mechanisms. The genetic diversity, population structure and distribution of strains indicate that transmission of *P. falciparum* was low, but that of *P. vivax* was still high in 2008. These data will be useful for assessing changes in malaria transmission resulting from interventions.

## Background

Temotu Province is the most southern province in the Solomon Islands. The province has a population of approximately 22,000, residing on five main groups of islands: Santa Cruz (Ndendo), Reef Islands, Duff Islands, Utupua and Vanikoro. Malaria is endemic in Temotu Province with year-round transmission. Historically malaria is highly prevalent in Temotu province. Blood slide examinations (through active and passive case detections) conducted in eastern districts, including Makira and Santa Cruz, in 1971 revealed a slide positive rate of 36.7% [1]. Expansion of the malaria eradication programme during 1972–1974, with activities including cyclical spraying operations with DDT and mass drug administration, lowered the slide positive rate rapidly to 3.2% in 1974 [1]. Since then malaria incidence rate has remained relatively low in Temotu Province. In 2008, when the Solomon Island government raised the goal of its national malaria programme from control to elimination, Temotu Province was consequently identified as the first province targeted for malaria elimination.

As a preliminary step towards elimination a baseline cross-sectional survey was conducted between October and November 2008 to obtain epidemiological information on malaria and vectors in the province. The blood survey covered approximately half of the population in the province and revealed an overall prevalence of *Plasmodium* spp. of 2.7% with *Plasmodium vivax* being the dominant species [2]. However, the parasite prevalence varied greatly between different islands with 11.6% on Duff Islands, 4.3% on Santa Cruz, and less than 2% on Utupua, Vanikoro and Reef Islands. The survey also revealed that 40 and 65.6% of microscopy-positive *Plasmodium falciparum-* and *P. vivax*-infected individuals respectively, had parasite densities below 100/μL, and that 83.7% of *P. falciparum* and 97.1% of *P. vivax*-infected subjects were asymptomatic at the time of survey and remained asymptomatic during the week of survey [3]. PCR detected a significant subset of individuals carrying sub-microscopic parasitaemia, predicting an overall point prevalence of 8.7% for the province [3]. The findings of the survey provided a good epidemiological baseline of the malaria situation and spatial distribution of infections in the province. The survey results also raised several important questions: what is the underlying cause of asymptomatic malaria in this setting? Are parasite populations on different islands different?

Asymptomatic malaria, often associated with low parasite densities, is usually observed in adults living in high transmission areas after repeated infections of malaria [4-9]. It correlates with the development of exposure-related clinical immunity to parasites. However, the high prevalence of asymptomatic malaria with low parasite density observed in Temotu Province occurred in areas of relatively low transmission intensity. Similar observations have been reported in other areas of low transmission, such as South America [10-15]. Asymptomatic malaria in these settings is difficult to explain by the relatively low number of exposures in this setting.

Clinical outcomes of malaria can be influenced by both parasite and host factors. Parasite factors include the pathogenesis of the parasite (density, cyto-adherence capability and pyrogenic threshold), the homogeneity of the parasite population and the relatedness of parasites within the population. While the pathogenesis properties of the parasite contribute directly to the clinical outcome of a patient, the homogeneity of parasite population and the relatedness of parasites influence the speed at which exposure-related and strain-specific immunity develops in the host population. It is possible that individuals living in low endemic areas develop clinical immunity after a small number of exposures and maintain the level of immunity due to low genetic diversity of the parasite population or close relatedness of the parasites. Host factors include erythrocyte polymorphisms, status of immune system and pregnancy.

In order to understand mechanisms underlying asymptomatic infections in Temotu Province, genetic diversity of both *P. falciparum* and *P. vivax* isolates collected during the 2008 baseline survey, their relatedness and distribution was analysed. The results shed light on the underlying cause of asymptomatic malaria in the province. From the malaria elimination aspect, asymptomatic malaria presents a major challenge because the large number of asymptomatic carriers can maintain malaria transmission and influence the transmission dynamics of malaria [6,16,17], and are difficult to diagnose microscopically or by rapid diagnostic tests (RDTs). These results will also serve as baseline information for measuring and evaluating impact of interventions and progress made towards elimination.

## Methods

### Study sites and sample collection

#### The 2008 baseline survey

**Figure 1:**
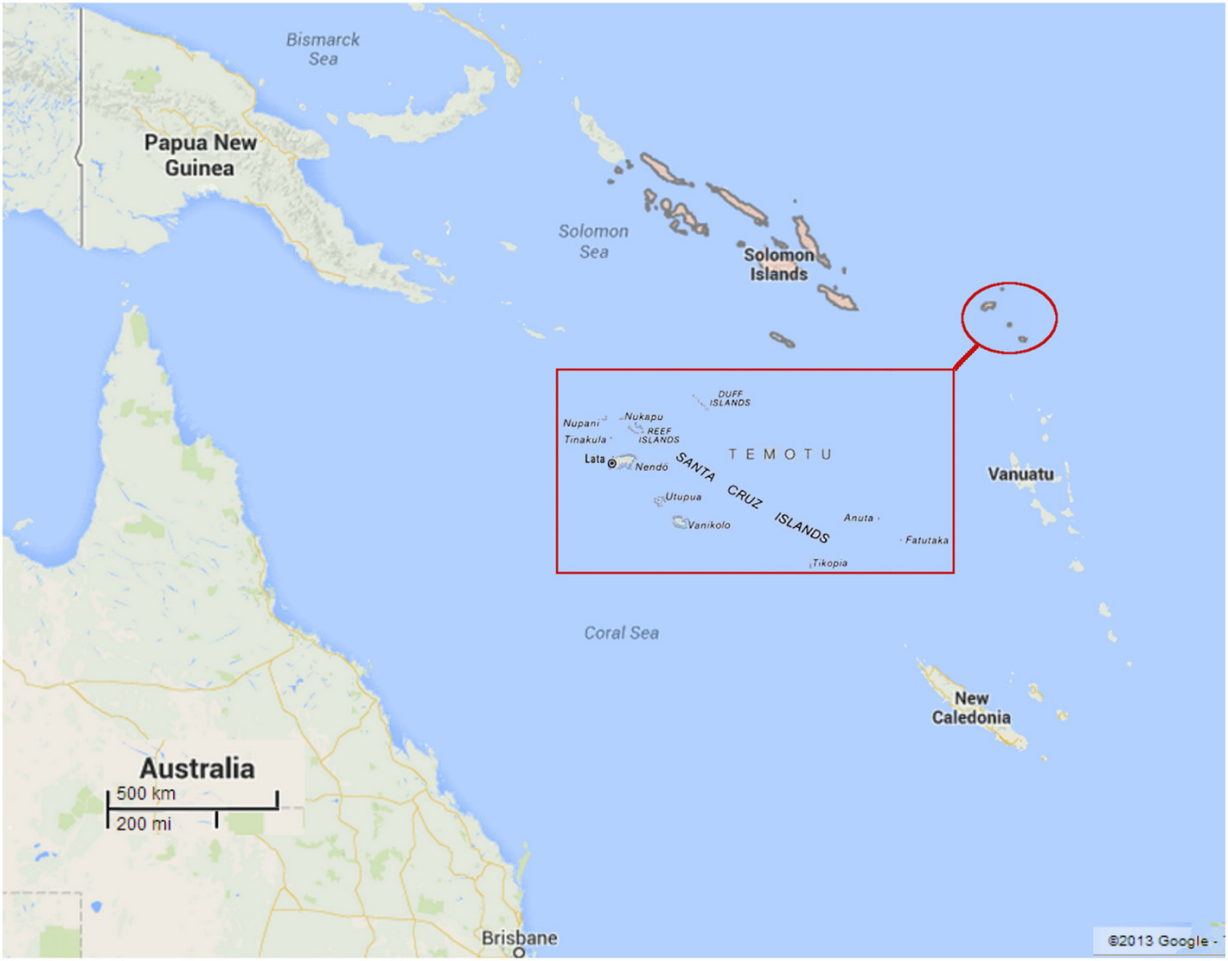
Map of Western Pacific region: geographical location of Solomon Islands and Temotu Province.

A mass blood survey was conducted in Temotu Province, Solomon Islands across the island groups of Santa Cruz (or Ndendo), Utupua, Vanikoro, Duff Islands and Reef Islands during October to November 2008 (Figure 1). The survey sites, consent process, sample collection methods, and malaria species detection and identification by microscopy are detailed elsewhere [2].

#### DNA extraction and PCR speciation

Extraction of genomic DNA from blood on filter papers and PCR to determine *Plasmodium* species were described previously [2]*.* DNA samples that were determined positive for *P. falciparum* and *P. vivax* were used for genotyping. Genomic DNA was also extracted from cultured *P. falciparum* lines of different origins and blood samples positive for *P. vivax* originating from other countries in the Pacific region, and used as positive and diversity controls for genotyping.

#### Microsatellite genotyping of Plasmodium falciparum

Eight microsatellite loci were genotyped using the primers described by Su and Wellems [18] and two round semi-nested PCR methods described by Anderson *et al.*[19] with some modifications to the PCR protocol including increasing cycle number by ten in each round. The microsatellite markers used were Polyα, TA1, TAA60, TAA81, TAA87, TAA109, ARA2 and PfPK2. Their chromosomal locations and fluorescent labelling are detailed in Table 1.

**Table 1:** **Summary of microsatellite markers, number of alleles, cumulative number of haplotypes and expected heterozygosity (***H*_E_**) at each marker for *Plasmodium falciparum *** **and *Plasmodium vivax *** **isolates collected in Temotu Province**

	**Locus**	**Chromosome location**	**Dye**	**n**	**No of alleles**	***H***_**E**_	**Cumulative number of haplotypes**
*Pf*	Polyα	4	PET	45	7	0.52	7
	PfPK2	12	NED	43	7	0.52	16
	TAA87	6	VIC	45	5	0.59	17
	TAA81	5	VIC	45	5	0.71	19
	TA1	6	6FAM	41	4	0.27	19
	TAA60	13	NED	44	4	0.55	19
	ARA2	11	PET	45	4	0.70	19
	TAA109	6	6FAM	44	3	0.46	19
	Mean ± SE			45	4.88 ± 0.51	0.54 ± 0.05	
*Pv*	MS8	12	VIC	60	20	0.94	20
	Pv3.27	3	6FAM	66	16	0.88	42
	MS16	9	NED	61	15	0.87	54
	MS10	13	PET	58	12	0.89	54
	MS5	6	NED	47	9	0.83	58
	msp1F3	7	VIC	62	7	0.79	58
	MS1	3	6FAM	48	5	0.75	58
	Mean ± SE			67	12 ± 2.02	0.85 ± 0.02	

For capillary electrophoresis, the second round labelled products were multiplexed in two combined dilutions: 1) Polyα, TA1, TAA60 and TAA81; and, 2) PfPK2, ARA2, TAA87 and TAA109, then run on an ABI 3100 Genetic Analyzer with POP7 at the QIMR Berghofer Medical Research Institute Scientific Services Analytic Facility. The length of PCR products was determined with reference to internal size standards ABI GS500 LIZ using Genescan and Peak Scanner software. Negative controls (no DNA and DNA from non-parasitised human blood) and standard strains (D6, W2 and TM96-C235) were included in each run for calibration between runs.

#### Microsatellite genotyping of Plasmodium vivax

Seven microsatellite markers were used to genotype *P. vivax* samples; this included three markers (Pv3.27, MS16 and *msp1*F3) that required a two round nested PCR approach [20] and four (MS1, MS5, MS8 and MS10) that were single round PCR [21]. Their chromosomal locations and fluorescent labelling are detailed in Table 1.

The primary round amplification was performed as a multiplex of all three genotyping markers in a reaction volume of 25 μL containing 3.2 mM MgCl_2_, 0.24 mM of each dNTP (0.96 mM total dNTPs), 0.2 μM of each primer, 2.5 μL of 10× ImmoBuffer and 0.625 units of Bioline Immolase™ DNA Polymerase with 1 uL of template DNA added. The thermocycling profile was as follows: initial denaturation of 95°C for 10 min, then 30 cycles of 95°C for 1 min, 56°C for 1 min, 72°C for 1 min, and final extension of 72°C for 5 min.

For the nested secondary round of amplification, each marker was amplified in a separate reaction in a volume of 25 μL using fluorescence labelled reverse primers. The reaction mix contained 3.2 mM MgCl_2_, 0.16 mM of each dNTP (0.64 mM total dNTPs), 0.2 μM of each primer, 2.5 μL of 10× ImmoBuffer and 0.625 units of Immolase. One μL of the first round product was added as template. The thermocycling profile was identical to the first round.

The additional four microsatellite markers (MS1, MS5, MS8 and MS10) were typed using the methods described by Gunawardena *et al.*[21].

Both sets of labelled products were diluted and pooled before being run on an ABI 3100 Genetic Analyzer at the QIMR Berghofer Medical Research Institute Scientific Services Analytic Facility. The length of PCR products was determined with reference to internal size standards ABI GS500 LIZ using Genescan and Peak Scanner software. Negative controls (no DNA and DNA from non-parasitised human blood) and positive controls (PVQ, AMRU1 and AMRU2) were used in every run.

#### Data analysis

Alleles were scored manually using Peak Scanner Software™ Version 1.0 (Applied Biosystems) using the height of 100 relative fluorescence units as the minimal peak threshold. The size range of the alleles and the average number of alleles per loci for each population were calculated.

#### Multiplicity of infection (MOI)

An infection was defined as polyclonal if there were two or more allele peaks present at one or more loci where the secondary peak was at least one-third the relative fluorescence unit height of the primary or highest peak [19]. MOI was calculated by dividing the total number of clones detected by the number of samples.

#### Allele and haplotype frequency

Allele frequency was calculated using only the predominant allele observed at each locus within each sample, while haplotype frequency was derived from combination of dominant allele type for each locus in each sample.

#### Expected heterozygosity (H_E_)

As a measure of genetic diversity *H*_E_ was determined for each marker, based on the allele frequencies, defined by *H*_E_ = [n/(n-1)][1-Σp_i_^2^] where n is the number of samples and p_i_ is the frequency of the *i*th allele, using FSTAT (Version 2.9.3.2) [22]. For samples with polyclonal infections, only the predominant allele was used for this calculation. *H*_E_ values for both *P. falciparum* and *P. vivax* populations were also calculated across all markers for Temotu Province and within island groups. For this and subsequent statistical analysis the samples were divided into two island groups: 1) Santa Cruz, 2) outer islands including Reef, Duff Islands, Utupua and Vanikoro.

#### Population differentiation (F_ST_)

Differentiation between populations, using pair-wise *F*_ST_, was calculated using FSTAT for comparing *P. falciparum* and *P. vivax* populations between the two island groups (Santa Cruz and outer islands including Reef, Duff Island, Utupua and Vanikoro) in Temotu Province.

#### Spatial distribution of haplotypes

Maps were generated using ESRI ArcMap9.2.

#### Relationships between parasite haplotypes

Phyloviz software [23] which implements the goeBURST algorithm [24], a refined version of the eBURST algorithm [25], was used to identify clusters of related haplotypes and generate a minimum spanning tree. Haplotype relationships were assessed using single locus variant (seven of the eight loci identical for *P. falciparum* and six of the seven loci identical for *P. vivax*) or double locus variant (six of the eight loci identical for *P. falciparum* and five of the seven loci identical for *P. vivax*).

## Results

### Genetic diversity

**Figure 2:**
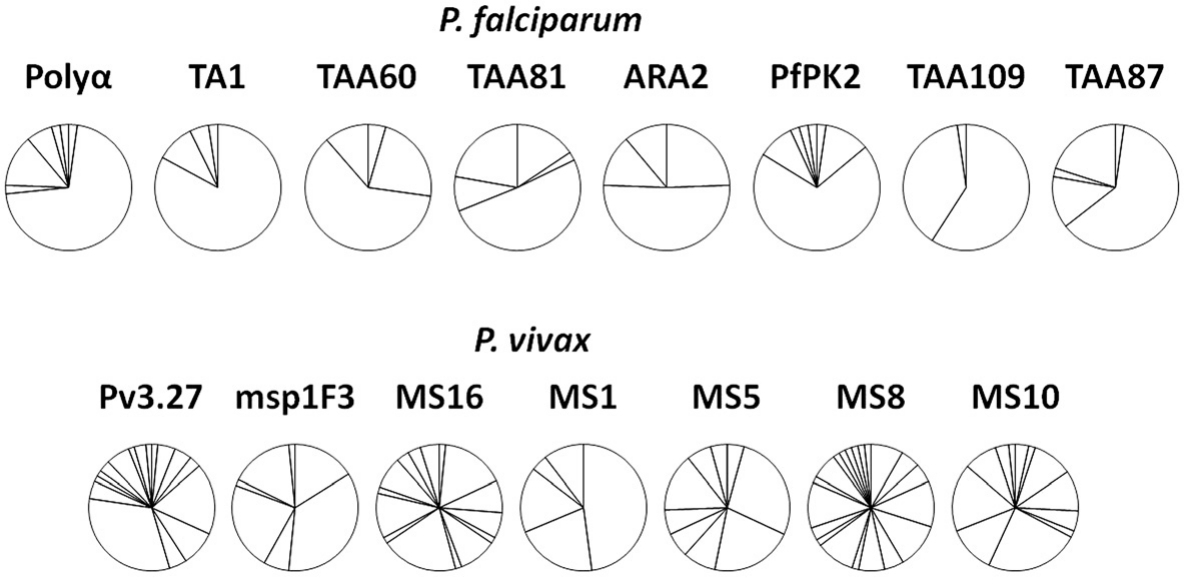
**The number of alleles and allele frequency of the microsatellite markers examined for *Plasmodium falciparum *** **and *Plasmodium vivax.***

**Figure 3:**
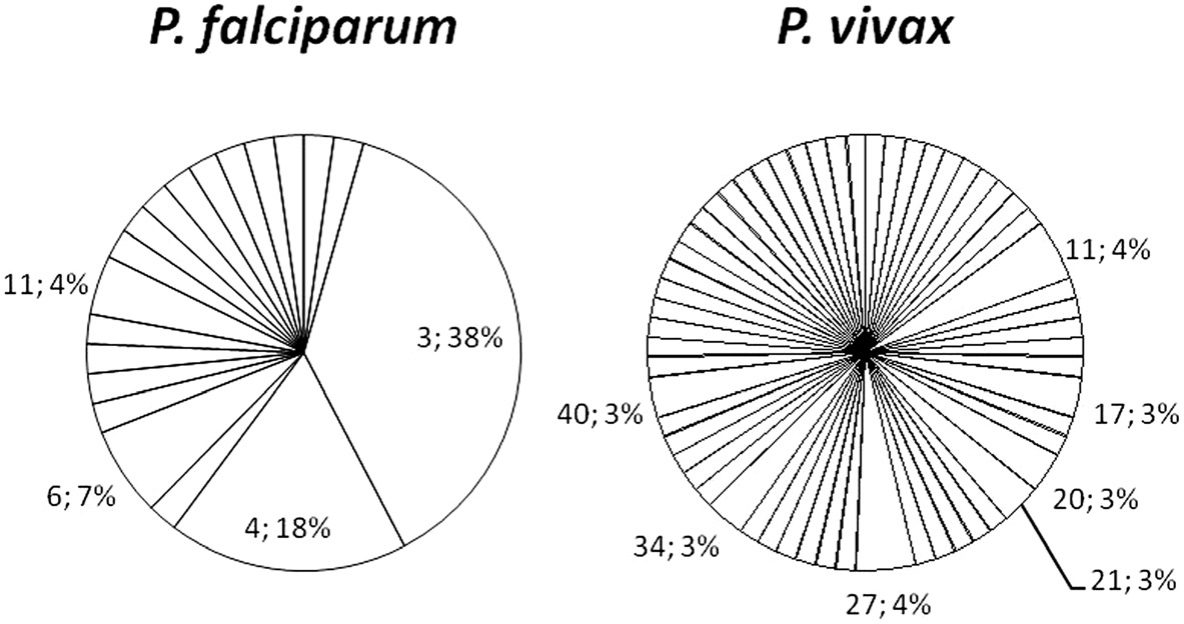
**The number of haplotypes and haplotype frequency for *Plasmodium falciparum *and *Plasmodium vivax.*** Only haplotypes with frequencies greater than 0.03, i e, >3%, are indicated in the figure (haplotype; percentage).

Overall, 8-loci-combined haplotypes were determined for 45 of *P. falciparum*. The median age of these subjects was 6.5 years (range from 1 to 60) with a median parasite density of 480/μL (range from 0 to 94,000/μL). The number of alleles per locus ranged from three to seven with two markers (Polyα and PfPK2) having the highest number of alleles (Table 1). Allele frequencies for each locus are illustrated in Figure 2. Combination of allelic types at four loci (Polyα, PfPK2, TAA87 and TAA81) produced a total of 19 haplotypes (PfH1-PfH19) from 45 *P. falciparum* samples examined (Table 1 and Figure 3). The number of haplotypes did not increase when four additional markers were typed (Table 1). Two haplotypes (PfH3 and PfH4) consisted of 38 and 18% of the *P. falciparum* population while the remaining 17 haplotypes made up 2-7% each. The expected heterozygosity (*H*_E_) for each locus ranged from 0.27 to 0.71 for *P. falciparum* with a mean of 0.54 (± 0.05, Table 1). There was no significant difference in *H*_E_ of *P. falciparum* between Santa Cruz and outer islands (Mann Whitney test, P = 0.55).

For *P. vivax*, 7-loci-combined haplotypes were determined for 67 samples. The median age of these subjects was 6 years (range from 0 to 30) with a median parasite density of 240/μL (range from 0 to 12,230/μL). The number of alleles per locus ranged from five for MS1 to 20 for MS8 (Table 1). MS8 also showed the most even distribution of different alleles with a highest allele frequency of 11.7% (Figure 2). Combination of allelic types at five loci (MS8, Pv3.27, MS16, MS10 and MS5) yielded a total of 58 haplotypes (PvH1-PvH58) in 67 samples examined. The number of haplotypes did not increase when additional two markers (MSP1F3 and MS1) were typed (Table 1). Of the 58 haplotypes, 51 (87.9%) were seen once, each making up only 1.5% of the *P. vivax* population (Table 1 and Figure 3). The most frequent haplotype was seen three times constituting 4.47% of the population. The *H*_E_ value for each locus ranged from 0.75 to 0.94 for *P. vivax* with a mean of 0.85 (± 0.02, Table 1). There is no significant difference in the *H*_E_ value for *P. vivax* between Santa Cruz and outer islands (Mann Whitney test, P = 0.55).

The overall *H*_E_ value of the *P. vivax* parasites was significantly higher than that of the sympatric *P. falciparum* parasites in Temotu Province (Mann Whitney test, P = 0.0003). The same is true for both Santa Cruz and the outer island group when tested separately (Table 2).

**Table 2:** **Comparison of***H*_E_**between Santa Cruz and outer islands in Temotu Province**

**Island groups**	***P. falciparum***		***P. vivax***		**p-value (Mann Whitney test)**
	***n***	***H***_**E**_	***n***	***H***_**E**_	
		**(Mean ± SE)**		**(Mean ± SE)**	
Santa Cruz	36	0.53 ± 0.04	45	0.84 ± 0.04	0.0012
Outer islands	9	0.56 ± 0.06	22	0.81 ± 0.03	0.0009
Total	45	0.54 ± 0.05	67	0.85 ± 0.02	0.0003

### Multiplicity of infection in *Plasmodium falciparum* and *Plasmodium vivax*

Two of the 45 *P. falciparum* isolates genotyped were two clone infections (detected on Polyα, TA1 and PfPK2) giving a MOI of 1.04. In contrast, 20 of the 67 *P. vivax* samples examined had greater than one clone on any of the seven markers examined (MOI = 1.33). The highest number of clones in one individual was three. While each of the seven markers detected at least one multiple clone infections, the combination of Pv3.27, msp1F3 and MS16 detected 80% (16/20) of the multiple clone infections in *P. vivax*.

### Shared haplotypes between island groups

**Figure 4:**
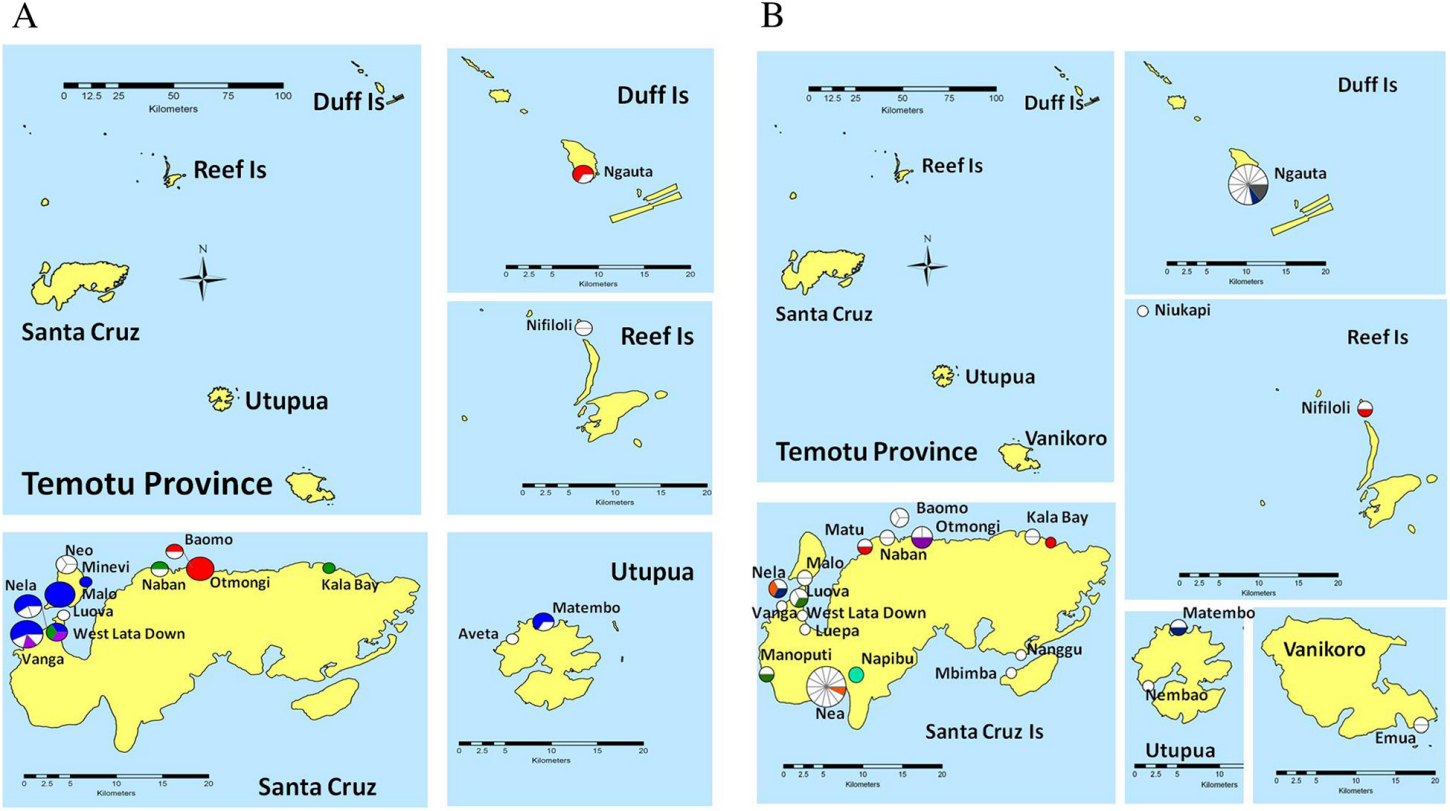
**Spatial distributions of *Plasmodium falciparum *(A) and *Plasmodium vivax *(B) haplotypes in Temotu Province.** A map of the Temotu Province is shown in the upper left panel while enlarged maps of particular islands are shown in the right and lower panel. Villages where parasites were genotyped are marked on the map. The type and frequency of haplotypes at each village are represented by a pie chart. The size of the pie chart is proportional to the number of parasites typed. White portions within pies indicate unique haplotypes observed once, while coloured portions indicate haplotypes observed more than once. Identical haplotypes are represented by the same colour. In Figure 4A, dominant *P. falciparum* haplotypes PfH3, PfH4, PfH6 and PfH11 are represented by blue, red, green and purple, respectively.

Two of the 19 *P. falciparum* haplotypes were observed on more than one island groups: PfH3, the most frequent haplotype, occurred on Santa Cruz (n = 15) and Utupua/Vanikoro (n = 2); PfH4 occurred on Santa Cruz (n = 6) and Duff /Reef Islands (n = 2) (Figure 4A). In contrast, only two of the 58 *P. vivax* haplotypes were shared between islands at the time of survey: PvH27 was observed once on Santa Cruz and twice on outer islands while PvH11 was observed once on Reef/Duff Islands and twice on Santa Cruz (Figure 4B).

### Differentiation of *Plasmodium falciparum* and *Plasmodium vivax* populations between island groups

The *F*_ST_ values for the *P. falciparum* and *P. vivax* populations in the two island groups are shown in Table 3. *F*_ST_ value between the two island groups for *P. falciparum* and *P. vivax* populations was below 0.033. The results indicate that both *P. falciparum* and *P. vivax* populations had low genetic differentiation between island groups. Therefore, there is no difference in parasite populations between Santa Cruz and outer islands in Temotu Province.

**Table 3:** ***F***_**ST **_**values top half of table (bold) and p-values after 1000 permutations bottom half of table**

**Population**	***P. falciparum***		***P. vivax***	
	**Santa Cruz**	**Outer islands**	**Santa Cruz**	**Outer islands**
Santa Cruz	-	**-0.016**	-	**0.033**
Outer islands	0.238	-	0.126	-

### Spatial distribution of *Plasmodium falciparum* and *Plasmodium vivax* haplotypes in Temotu Province

*Plasmodium falciparum* appears to cluster in the northwestern side of Santa Cruz Island. Three dominant haplotypes PfH3, PfH6 and PfH11, were all identified in western coastal villages indicating a focal transmission (Figure 4A). PfH4 was also seen in two northern villages on Santa Cruz Island and the southern tip of Duff Island. In contrast, *P. vivax* was identified on both the north and south coast of Santa Cruz and on all other islands. Unique haplotypes, most identified only once, were scattered in different villages (Figure 4B).

### Genetic relatedness among haplotypes

**Figure 5:**
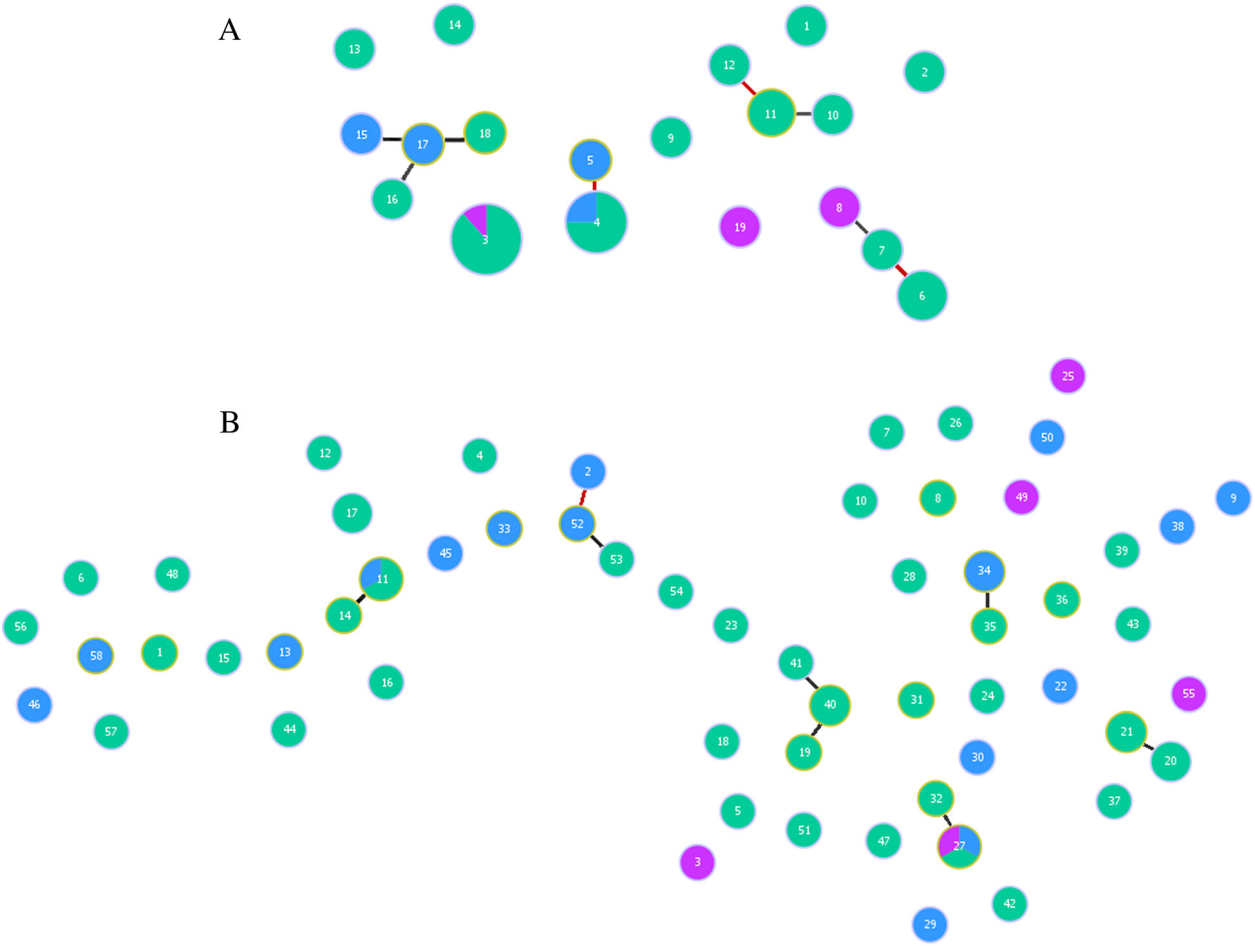
**Relationships among *Plasmodium falciparum *(A) and *Plasmodium vivax *(B) haplotypes in Temotu Province.** Each ball represents a haplotype and the size is proportional to the number of samples with that haplotype. Colour of the ball represents different island groups: blue, green and purple represent Reef/Duff Islands, Santa Cruz and Utupua/Vanikoro, respectively. Red lines highlight single locus variants (seven of eight loci identical for *P. falciparum* and six of seven loci identical for *P. vivax*) while black lines highlight double locus variants (six of eight loci identical for *P. falciparum* and five of seven loci identical for *P. vivax*).

In *P. falciparum* haplotypes, four closely related clusters were identified: three clusters were formed by two single locus variants (identical on seven of the eight loci) and one cluster was formed by four double locus variants (identical on six of the eight loci). Each of the single locus variant clusters included one haplotype consisting more than 4% of the *P. falciparum* population indicating clonal expansion of these clusters. Two of these small clusters were observed on Santa Cruz, one shared between Santa Cruz and the Duff Islands, and one shared between Santa Cruz and the Reef Islands (Figure 5A).

For *P. vivax*, only one small cluster of single locus variant and five small clusters of double locus variants were identified. The single locus variant cluster contained two haplotypes that were each seen only once on the Duff Islands (Figure 5B). The five double locus variants, each contain two to three haplotypes, were mostly seen in Santa Cruz (Figure 5B).

### Relationship between haplotype and clinical and parasitological features

Of the 45 *P. falciparum* samples analysed, eight were collected from febrile patients. From these symptomatic patient samples haplotype PfH3 was predominant (in six patients) and was significantly associated with fever (Fisher's exact test, P = 0.0388). It was followed by PfH4 and PfH15, in one patient each. PfH3 was also associated with high parasite densities as 29% (5/17) patients with PfH3 had parasite densities ≥10,000/μL, compared to 3.6% (1/28) in non-PfH3 haplotypes (Fisher’s exact test, P = 0.028). Age of individuals infected with PfH3 parasite (known for 13/17 individuals) ranged from five to 56, with a mean of 24.54 (± 4.21).

## Discussion

Temotu Province, Solomon Islands is progressing towards malaria elimination. To better understand malaria epidemiology in this setting a baseline survey was conducted in 2008. The survey showed that most *Plasmodium* infections in the province were of low parasite density and asymptomatic infections [3]. The current study aims to examine genetic diversity and relationships among *P. falciparum* and *P. vivax* populations in Temotu Province in order to shed light on the mechanism underlying the observed malaria transmission characteristics in this setting.

A total of 45 *P. falciparum* and 67 *P. vivax* samples collected in the 2008 baseline study were analysed in this study. Like other epidemiology studies there is some unavoidable bias against samples with very small volume or very low parasite densities because of difficulties in amplifying markers at multiple loci. Parasite density and subject’s age appeared to be two major determinants for the success of genotyping on 7 or 8 markers. The median parasite density in samples successfully genotyped were 480 and 240 parasites/μL compared to 10 and 16 parasites/μL, for *P. falciparum* and *P. vivax*, respectively, in those could not be genotyped (Mann Whitney test, P < 0.0001). The median age of subjects successfully genotyped were significantly younger than subjects not genotyped for both *P. falciparum* (6.5 vs 14, Mann Whitney test, P = 0.02) and *P. vivax* (6 vs 9, Mann Whitney test, P = 0.002).

Genetic diversity among the *P. falciparum* and *P. vivax* populations in Temotu Province was compared by measuring diversity on eight and seven microsatellite markers, respectively. Most of these markers are neutral, located on different chromosomes, and have been widely used for population genetics studies [21,26,27]. For the *P. falciparum* population, results revealed 19 haplotypes in 45 samples analysed giving an average *H*_E_ of 0.54, moderately diverse. The level of diversity for *P. falciparum* in Temotu Province is comparable to that reported in Thailand, higher than that in South America, but lower than that in Papua New Guinea (PNG) and African *P. falciparum* populations in 1990s [26] using similar but a larger set (12) of microsatellite markers.

In contrast, the *P. vivax* population was highly diverse. A total of 58 haplotypes were obtained in 67 *P. vivax* samples, giving an average *H*_E_ of 0.85, which was significantly higher than that in the sympatric *P. falciparum* population in the province, despite using one less marker (seven instead of eight). This high level of diversity is comparable to that reported in PNG [28], Tetere area, Solomon Islands [29], Kolkata [30], Sri Lanka and Myanmar [21], and higher than that reported for India, Thailand, Laos, Colombia [27] and Ethiopia [21] using similar markers. The findings are similar to that reported in Brazil where *H*_E_ for *P. vivax* and *P. falciparum* was determined to be 0.80 and 0.51, respectively [31].

The *P. falciparum* parasites in the province showed some evidence of clonal expansion with four closely related clusters representing 48.5% of *P. falciparum* population. These clusters, plus the most dominant haplotype PfH3 (38%), represented 86.5% of the *P. falciparum* population in Temotu Province at the time of survey. Moreover, there was no evidence of a major bottle neck resulting in a loss of rare allele since 89% (17/19) of the haplotypes observed had a frequency below 0.1. In contrast, there was no evidence for clonal expansion in the *P. vivax* population. Only six small closely related clusters, involving 14 of the 58 haplotypes, were identified, representing only 33% of the *P. vivax* population.

Genetic diversity in parasite population is closely associated with transmission intensity of an area. High levels of genetic diversity are usually seen in areas with high levels of transmission where multiple clone infections are common, while as low diversity or clonal populations could be seen in areas with low transmission [32]. Genetic diversity levels observed in this study would suggest that the transmission intensity of *P. falciparum* in Temotu was low, but the transmission intensity of *P. vivax* was still high in 2008. This is in agreement with the prevalence data obtained during the survey where prevalence was 1.3 and 2.1% for *P. falciparum* and *P. vivax*, respectively [2]. This is also supported by higher MOI of *P. vivax* than *P. falciparum* (1.33 *vs* 1.04). While this difference in diversity between species could have resulted from differential selection in the history or differential susceptibility [33] of the only vector in the province, *Anopheles farauti*[34], it is also possible that the intervention measures were more effective in reducing transmission of *P. falciparum* than *P. vivax,* largely due to difficulties in curing the latent hypnozoite stage of *P. vivax*. The high diversity in *P. vivax* population is likely maintained by a large number of people carrying hypnozoites in their liver.

Although a high rate of asymptomatic malaria was associated with both *P. falciparum* (83.7%) and *P. vivax* (97.1%) infections [3], the underlying mechanism is likely to be different between the two species. The *P. falciparum* population in Temotu Province has relatively low genetic diversity and parasites appear to be genetically closely related. This could speed up the development of acquired immunity to the *P. falciparum* population and thereby partially explain the high proportion of asymptomatic *P. falciparum* infections. In contrast, the *P. vivax* population was found to be highly diverse and not closely related. The large proportion of asymptomatic vivax malaria observed in the province could not be explained by lack of genetic diversity or the relatedness of the parasite population. This phenomenon has been increasingly reported in several areas with low transmissions [12-15]. It is possible that clinical immunity can be maintained with limited exposure and/or with prolonged infection at low parasite density; alternatively, other host factors may play a role in this phenomenon.

This study also provides important information for the malaria elimination programme in Temotu Province. Firstly, there was no significant differentiation of the parasite populations between Santa Cruz and the outer islands, and parasite haplotypes seen more than once were frequently found shared between different islands. These results suggest that there was relatively free movement of people carrying parasites among these islands. The provincial malaria elimination interventions would be more effective if coverage is for all islands.

Secondly, the study provides evidence for possible importation of *P. falciparum* strains. The PfH3 haplotype was the most dominant strain in the province consisting of 38% of the *P. falciparum* population. This strain was observed mostly in the western villages of Santa Cruz indicating focal transmission. PfH3 was significantly associated with fever as well as with high parasite densities. The focal distribution and illness associated with this strain suggest that the local host population lacks immunity against this strain of *P. falciparum.* This is further supported by the wide age range of the hosts infected with this strain, instead of predominantly children. Combined, these data indicate that PfH3 may be a newly imported parasite. This finding suggests that to eliminate malaria, vigilance should be maintained to prevent importation of parasites from other malaria areas.

Finally, this study provides baseline genetic diversity data for both *P. falciparum* and *P. vivax* populations in Temotu Province. Comparing future survey findings against these baseline data will help to assess any changes in parasite strains, genetic diversity and population structure. As these parameters are associated with malaria transmission intensity they will be useful for evaluating the impact of interventions and progress toward elimination.

## Conclusion

The low diversity and clonal population of *P. falciparum* population may partially account for the high level of clinical immunity. However, it is possible that importation of a new *P. falciparum* strain has caused most of the symptomatic cases. In contrast, high diversity in the *P. vivax* population and low relatedness between strains suggested clinical immunity to *P. vivax* may be maintained by different mechanisms such as requiring limited exposures, prolonged infections with low parasite densities or host factors, rather than population homogeneity. The genetic diversity, population structure and distribution of strains indicate that transmission of *P. falciparum* was low, but that of *P. vivax* was still high in 2008. These data will be useful for assessing changes in malaria transmission resulting from interventions.

## Competing interests

The authors declare that they have no competing interests.

## Authors’ contributions

KG, SD, and LB performed experiment determining parasite genotypes; KG, SD, LB and QC carried out data analysis; AB and LW contributed to the baseline survey; KG and QC wrote the manuscript; KG, DS and QC conceived and designed the study. All authors read and approved the final manuscript.
